# Employees and the National Insurance Act

**Published:** 1912-08-03

**Authors:** 


### OUR NATIONAL INSURANCE ACT DEPARTMENT.

### EMPLOYEES AND THE NATIONAL INSURANCE ACT.

### XXII.—Substituted Benefits.

One of the principal grounds on which criticism of the National Insurance Act has been focussed is its too rigid adherence to a standard type both for contributions and benefits. This is an inherent difficulty in any scheme drafted on national lines. Conditions of life in different parts of the country are so diverse, and the requirements of different classes of people are so dissimilar that it would be wellnigh impossible to devise a scheme that would be agreeable to all. It was, of course, recognised that the National Insurance scheme would have to be modified in the case of several special classes of persons, and provision is made for these special classes in the ten sections of the Act numbered 44 to 53 inclusive. These special provisions extend to (1) married women, (2) aliens, (3) persons in the naval and military service of the Crown, (4) persons in employment where the employer is liable to pay wages during sickness, (5) persons in the mercantile marine, (6) persons who were over sixty-five years of age when the Act came into operation, (7) persons who are engaged in trades of a seasonal character, (8) inmates of charitable homes, (9) persons becoming certificated teachers, and (10) other persons in the service of the Crown. Although this is a very full list, it by no means exhausts the number of special classes to whom the standard benefits and contributions are not suited to their requirements and means. Fortunately for these other special classes of persons it is possible to obtain some modification of the standard benefits under the operation of Section 13.

#### Schemes must be Approved by the Commissioners.

By this section it is provided that any approved society may submit to the Insurance Commissioners a scheme for substituting any of the additional benefits, as specified in Part II. of the Fourth Schedule to the Act, for sickness and disablement benefits or either of them, or for any part of such benefits. The scheme need not apply to the whole of the members of the society as it may only be desired that it should apply to a specified class, or to those members who elect to come under the scheme. It is, however, important to note that no such scheme can be adopted until the formal approval of the Commissioners has been given, and, further, that it is laid down in the Act that this consent is not to be given by the Commissioners unless they are satisfied there are good grounds for allowing the scheme for substituted benefits, in view of the special circumstances of the class of members to whom it is to apply.

As the matter was one which demanded close attention from the Commissioners, a special sub-committee was appointed by the Joint Committee of the respective Commissions to undertake the duty of preparing alternative schemes, together with actuarial tables, in order that societies which propose to take advantage of the section may have sufficient guidance to enable them to consider the question. The report of this sub-committee was published in the form of a Blue-book quite recently, together with tables of rates of pension allowances that can be given in place of the sickness and disablement benefits relinquished.

#### An Important Warning.

The publication is prefaced by a special introduction signed by Mr. C. F. G. Masterman, Chairman of the Joint Committee, in which the following passage occurs:—"The Joint Committee, as at present advised, are of opinion that the schemes comprised in the report will be suitable only in the case of those insured persons who, by the nature of their employment, are able to look forward with some assurance to long and regular employment, and to receiving during sickness from their employers benefits equivalent to those provided under the Act under the head of sickness benefit. The Committee have agreed that these conditions are met substantially in the case of certain classes of teachers, nurses, clerks, shop assistants, and domestic servants, and the Commissioners will be prepared to consider schemes applicable to such classes and to other classes in respect of which the like conditions can be shown to obtain. But even among these classes it will be a matter for serious consideration by each individual whether in his special circumstances the substituted benefit is more suitable than that which is to be surrendered, and for this purpose it is hoped that the employers of domestic servants in particular will assist their servants in considering the question."

This is a most important warning which should be given full consideration by every individual who has any thought of adopting either one or other of the substituted benefits.

#### The Official Schemes.

The schemes suggested by the sub-committee and adopted by the Joint Committee of the Commissioners provide for pensions through a superannuation fund. There are several alternative plans. In the first place, the pensions may be taken in substitution for sickness benefit alone, without relinquishing the right to disablement benefit until the attainment of the age of sixty-five, and the whole of the sickness benefit and half disablement benefit from the age of sixty-five to the age of seventy. In the second place, the pensions may be substituted for the whole of the sickness and disablement benefits throughout insurance. For each of these alternative plans there are, in the case of men, three ways in which the pensions may be taken. The pension in all cases commences at the age of sixty-five, and the variations are in regard to the time for which they will continue to be payable. The first table shows the rates of pension per week payable throughout life. The second shows the amount of pension per week beginning at the age of sixty-five and ending at the age of seventy, while the third shows the weekly pension payable throughout life commencing at the age of sixty-five, with the special provision that the amounts of pension are to be reduced by 5s. a week at the age of seventy. This will be made clearer by means of an extract from the table.

Pension at Age of Sixty-five (Men).

| Age last birthday at entry into scheme. | Pension in substitution for Sickness and Disablement Benefits. | | | | | |
|---|---|---|---|---|---|---|
| | Throughout life from age 65. | | Beginning at age 65 and ending at age 70. | | Beginning at age 65 and reduced by 5/- a week at age 70. | |
| | Per week s. | d. | Per week s. | d. | Per week s. | d. |
| 16 | 5 | 9 | 12 | 1 | 8 | 4 |
| 25 | 4 | 9 | 9 | 11 | 7 | 4 |
| 35 | 3 | 9 | 7 | 10 | 6 | 4 |
| 45 | 2 | 9 | 5 | 9 | 5 | 4 |

In the full table rates are given for each at entry upon the scheme up to the age of forty-five. It is important to note, however, that the rates as here set forth are the full amounts obtained by regular contribution, and they will, therefore, be subject to reduction if the contributions are allowed to fall into arrear. In calculating the arrears the usual allowance is made for contributions not falling due when the insured person is entitled to sickness or disablement benefit. It must also be pointed out that the contributions will continue to be payable until the age of seventy. The amounts of pension include the State proportion of two-ninths.

The corresponding table for men when the benefit surrendered is sickness benefit throughout insurance and half disablement benefit from sixty-five to seventy shows pension payments considerably less in amount than those in the foregoing table. There is a special reason for offering the pension in the form of an annuity payable throughout life but reducible by 5s. a week at the age of seventy, because on the attainment of that age the insured person will be entitled, if his means do not exceed £21 a year, to a pension of 5s. a week under the National Old Age Pension Act, 1908. In the case of women this form of pension cannot be granted, because at the youngest age at entry into the scheme, namely, age of sixteen last birthday, the amount of the weekly pension payable throughout life commencing at the age of sixty-five is only 4s. 1d., whilst at higher entry ages the amount of pension that can be granted is necessarily less than this.

Pensions for Women.

There are two reasons why the women's pensions are less than those that can be granted to men. The first reason is that the amount of sickness benefit to which they are entitled under the Act is 7s. 6d. a week instead of 10s., and therefore the amount of sick pay relinquished is smaller. The second reason is that women live longer than men on the average, so that for the same amount of contribution at, say, the age of twenty, the amount of pension that could be given on the attainment of the age of sixty-five in the case of a woman would be less than to a man.

Further tables are provided which show the amounts of pension which are purchased by each successive year of sickness benefit forgone, by means of which it will be possible to calculate the variations in the amount of the pension payments should contributions fall into arrear, and a further use to which they may be put is the calculation of the pension already purchased in the event of a member of an approved society who has adopted the scheme wishing to revert to the ordinary benefits under the Act.

A Popular Feature.

It is by no means certain that these substituted pensions will meet with general acceptance, even though there may be no necessity for the sickness and disablement benefits, because the provision for benefits that cannot be entered upon until the age of sixty-five is not popular. There is, however, one popular feature of the proposed scheme as it affects women, namely, that it is recommended that a woman should be given the option on or after marriage to receive a cash surrender value of three-eighths of the contributions paid in respect of her with interest, that is to say, the amount derived from her own contribution to the superannuation fund as distinct from the amount derived from the contribution of the employer and the State. As sickness rights at the earlier ages are worth about 6s. a year and disablement rights at these ages have practically no actuarial value, the amount of this cash surrender will in the case of women marrying early in life be about 2s. 3d. for each year of contribution to the fund. In the case of women who, on passing out of insurance, have not contributed to the fund five years, and do not elect to become voluntary contributors at the special reduced rate, the remaining five-eighths will enure to the benefit of the superannuation fund. Women who have contributed to the fund for five years or upwards may receive the cash surrender value of three-eighths of the contributions, and in such case the remaining five-eighths will be carried to a suspense account, and will be paid as a gratuity on attainment of the age of sixty-five with the accumulation of interest earned during the period of suspension.

It is the intention of the Commissioners that the amount of the pension should in no case be less than 2s. 6d. a week, and where the contributions to the fund have not been sufficient to enable this pension to be paid the value of the pension payment that has been provided will be commuted for a single cash sum payable at the age of sixty-five.

It should be noted that the substitution of pension benefit for sickness and disablement allowances does not affect the right of the insured person to medical, sanatorium, or maternity benefit.

---

NOTE.—Questions and Correspondence on all doubtful points are invited, and should be addressed to the Editor, The Hospital, 28 Southampton Street, Strand, W.C., and marked "Insurance."

